# Supraclavicular lymph node incisional biopsies have no influence on the prognosis of advanced non-small cell lung cancer patients: a retrospective study

**DOI:** 10.1186/s12957-016-1064-5

**Published:** 2017-01-09

**Authors:** Song Dong, Ning Zhao, Wei Deng, Hui-wen Sun, Fei-yu Niu, Jin-ji Yang, Wen-zhao Zhong, Feng Li, Hong-hong Yan, Chong-rui Xu, Qiu-yi Zhang, Xue-ning Yang, Ri-qiang Liao, Qiang Nie, Yi-long Wu

**Affiliations:** 1 Guangdong Lung Cancer Institute, Guangdong General Hospital & Guangdong Academy of Medical Science, 106 Zhongshan 2nd Road, Guangzhou, 510080 People’s Republic of China; 2 Department of Thoracic Surgery, The First People’s Hospital of Foshan, Foshan, People’s Republic of China; 3 Southern Medical University, Guangzhou, People’s Republic of China

**Keywords:** Non-small cell lung cancer, Supraclavicular lymph node, Incisional biopsy

## Abstract

**Background:**

Supraclavicular lymph node (SCLN) biopsies play an important role in diagnosing and staging lung cancer. However, not all patients with SCLN metastasis can have a complete resection. It is still unknown whether SCLN incisional biopsies affect the prognosis of non-small cell lung cancer (NSCLC) patients.

**Methods:**

Patients who were histologically confirmed to have NSCLC with SCLN metastasis were enrolled in the study from January 2007 to December 2012 at Guangdong Lung Cancer Institute. The primary endpoint was OS, and the secondary endpoints were complications and local recurrence/progression.

**Results:**

Two hundred two consecutive patients who had histologically confirmed NSCLC with SCLN metastasis were identified, 163 with excisional and 39 with incisional biopsies. The median OS was not significantly different between the excisional (10.9 months, 95% CI 8.7–13.2) and incisional biopsy groups (10.1 months, 95% CI 6.3–13.9), *P* = 0.569. Multivariable analysis showed that an Eastern Cooperative Oncology Group (ECOG) performance status (PS) ≥2 (HR = 2.75, 95% CI 1.71–4.38, *P* < 0.001) indicated a worse prognosis. Having an epidermal growth factor receptor (*EGFR*) mutation (HR = 0.58, 95% CI 0.40–0.84, *P* = 0.004) and receiving systemic treatment (HR = 0.36, 95% CI 0.25–0.53, *P* < 0.001) were associated with a favorable OS. Neither the number (multiple vs. single) nor site (bilateral vs. unilateral) of SCLNs was associated with an unfavorable OS, and SCLN size or fixed SCLNs did not affect OS.

**Conclusions:**

SCLN incisional biopsies did not negatively influence the prognosis of NSCLC patients. It was safe and feasible to partly remove a metastatic SCLN as a last resort in advanced NSCLC.

## Background

Lung cancer threatens human health greatly, and patients who present with supraclavicular metastasis are classified as stage IIIB or IV and are therefore not considered candidates for surgical treatment [1]. Supraclavicular lymphadenectomy plays an important role in diagnosing and staging lung cancer. There were no evidences on the survival advantage of excision on supraclavicular lymph nodes in advanced lung cancer patients. The advantage of lymphadenectomy is that we could get the accurate N stage. Besides, it provides more tissue than a needle biopsy for detection of gene profile with less complication. In clinical practice, we prefer to perform complete excision because of the tumor-free principle. Unfortunately, complete excision cannot be performed on all patients. Partial removal of the tumor is inevitable when metastatic lymph nodes are too large, in an inaccessible location, or when they tightly adhere to important adjacent structures with unclear margins.

Over the past decades, the question of whether an incisional biopsy affects the prognosis of melanoma patients has been very controversial, with evidence suggesting there appears to be little difference [2–4]. For early stage lung cancer, radical lung resection combined with systematic nodal dissection is recommended, and is associated with a better prognosis [5, 6], while incomplete resection (R1/R2) is associated with a higher incidence of local recurrence and a worse prognosis [7–9], possibly because of cancer cell seeding and dissemination during the process of incomplete resection. However, prognoses are different for advanced lung cancer. Metastasectomy or other local therapy in malignancy was often used in certain situations, and the major goal of local treatment was debulking rather than eradication [10]. Though supraclavicular lymph node (SCLN) resection as a biopsy method was widely used in practice; there were few reports about whether partial removal of metastatic SCLNs would lead to a worse prognosis in lung cancer, which is of concern for both doctors and patients.

The aim of the current study was to explore the influence of SCLN incisional biopsy on non-small cell lung cancer (NSCLC) prognosis.

## Methods

### Patients

Patients who were histologically confirmed with NSCLC with SCLN metastasis were enrolled into the study from January 2007 to December 2012 at Guangdong Lung Cancer Institute (GLCI). Patient eligibility criteria were as follows: (1) Histological confirmation of NSCLC. (2) Confirmation of metastatic NSCLC through supraclavicular lymphadenectomy. (3) No other concomitant malignancies. The ethics committee of Guangdong General Hospital approved the study, and informed patient consent was waived because of the retrospective nature of the study. Statisticians acquired follow-up information every 3 months from the registries of the GLCI.

### Supraclavicular lymphadenectomy

SCLNs were diagnosed using palpation or imaging at the first visit and then histologically confirmed after resection. Three residents performed the supraclavicular lymphadenectomy following the principles below: First, metastatic lymph nodes should be completely removed. Second, one or more lymph nodes should be removed with intact capsule when multiple lymph nodes were enlarged and removing all of them was not feasible. Either complete resection or intact removal of one of several lymph nodes was defined as an excisional biopsy. Third, an incisional biopsy should be performed only as a last resort when an excisional biopsy was impossible.

### Statistical analysis

Pearson’s chi-square test was used to compare the frequency of the baseline data. The Kaplan–Meier method was used to estimate survival outcomes, and the difference in survival was compared using the log-rank test. Multivariable Cox regression was used to evaluate independent prognostic factors associated with overall survival (OS). *P* < 0.05 was deemed to be statistically significant. All *P* values and 95% confidence intervals (95% CI) were two-sided. Statistical analyses were done using the Statistic Package for the Social Sciences (SPSS, version 13.0). The primary endpoint was OS, which was defined as the interval from initial pathologic diagnosis to death. The secondary endpoints were complications and local recurrence/progression. Local recurrence/progression was defined as relapse of lymph nodes on the biopsy side in the excisional biopsy group or obviously enlarged in the incisional biopsy group after biopsy.

## Results

From January 2007 to December 2012, we identified 202 consecutive histologically confirmed NSCLC patients with SCLN metastasis, 163 had excisional and 39 had incisional biopsies. Baseline demographic and clinical characteristics are shown in Table 1. The median age was 58 years (range 26–85). Most patients (164/202, 81.2%) were stage IV at initial diagnosis and were diagnosed with adenocarcinomas (169/202, 83.7%). Patients receiving an incisional biopsy were more likely to be male (*P* = 0.003), and a current or ever-smoker (*P* = 0.008), with larger lymph node size (*P* < 0.001) and fixed lymph nodes (*P* < 0.001).

**Table 1:** Baseline demographic and clinical characteristics

Characteristics	Excisional biopsy	Incisional biopsy	Total	*P* value
	(*n* = 163)	(*n* = 39)	(*n* = 202)	
Sex				0.003
Male	92 (56.4%)	32 (82.1%)	124 (61.4%)	
Female	71 (43.6%)	7 (17.9%)	78 (38.6%)	
Age				0.922
Median	58 (26–85)	58 (29–75)	58 (26–85)	
<65	110 (67.5%)	26 (66.7%)	136 (67.3%)	
≥65	53 (32.5%)	13 (33.3%)	66 (32.7%)	
Smoking status				0.008
Never-smoker	93 (57.1%)	13 (33.3%)	106(52.5%)	
Current/ever-smoker	70(42.9%)	26(66.7%)	96 (47.5%)	
ECOG PS				1.000^a^
PS 0–1	143 (87.7%)	35 (89.7%)	178 (88.1%)	
PS ≥2	20 (12.3%)	4 (10.3%)	24 (11.9%)	
Histology				0.747
Adenocarcinoma	138 (84.7%)	31 (79.5%)	169 (83.7%)	
Squamous	13 (8.0%)	4 (10.3%)	17 (8.4%)	
Large cell	7 (4.3%)	2 (5.1%)	9 (4.5%)	
Other NSCLC	5 (3.1%)	2 (5.1%)	7 (3.5%)	
Stage				0.448
IIIB	29 (17.8%)	9 (23.1%)	38 (18.8%)	
IV	134 (82.2%)	30 (76.9%)	164 (81.2%)	
LN size				<0.001
Mean size	18.6 mm	25.9 mm	20.0 mm	
>2 cm	34 (20.9%)	23 (59.0%)	57 (28.2%)	
≤2 cm	129 (79.1%)	16 (41.0%)	145 (71.8%)	
LN site				0.107
Unilateral	132 (81.0%)	27 (69.2%)	159 (78.7%)	
Bilateral	31 (19.0%)	12 (30.8%)	43 (21.3%)	
LN number				0.109
Single	77 (47.2%)	24 (61.5%)	101 (50.0%)	
Multiple	86 (52.8%)	15 (38.5%)	101 (50.0%)	
Fixed LN				<0.001
Fixed	119 (73.0%)	6 (15.4%)	125 (61.9%)	
Unfixed	44 (27.0%)	33 (84.6%)	77 (38.1%)	
Systemic treatment				0.141
Yes	127 (77.9%)	26 (66.7%)	153 (75.7%)	
No	36 (22.1%)	13 (33.3%)	49 (24.3%)	

*ECOG PS* Eastern Cooperative Oncology Group performance status, *LN* lymph node

^a^Fisher’s exact test

Most patients (86.1%, 174/202) were treatment-naïve at the time of biopsy. Of the patients, 75.7% (153/202) received systemic treatment; 77.9% in the excisional vs. 66.7% in the incisional biopsy group. There was no significant difference in receiving systemic treatment between the two groups; *P* = 0.141. We noted that 49 patients (24.3%) had no record of receiving any antitumor treatment during their lifetimes, in most cases (57.1%) because of pessimistic attitude or financial problems and in nine cases (18.4%) because of poor performance status (PS). Twenty-three patients (11.4%) had received SCLN radiotherapy before or after resection, and most of them had stage IIIB disease (69.6%, 16/23). The percentages of patients receiving radiotherapy in the excisional and incisional biopsy groups were 11.7 (19/163) and 10.3% (4/39), respectively.

**Fig. 1:**
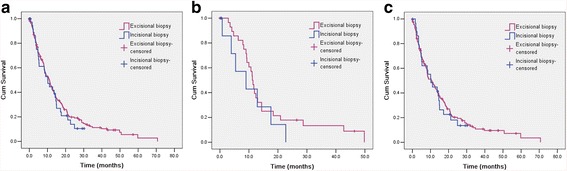
**a** Kaplan–Meier curves of overall survival in all patients, **b** overall survival in stage IIIB patients, **c** and overall survival in stage IV patients

The median follow-up time was 9.3 months. At the final follow-up date, May 11, 2014, 171 (84.7%) patients had died. The median OS was 10.7 months (95% CI 8.7–12.7 months) for all patients. There was no significant difference between the excisional (OS = 10.9 months, 95% CI 8.7–13.2) and incisional biopsy groups (OS = 10.1 months, 95% CI 6.3–13.9), *P* = 0.569 (Fig. 1a), and the 1-year survival rates, 46.0 vs. 47.0%, respectively. In subgroup analysis, OS did not show any difference between the two groups in either stage IIIB (11.2 vs. 9.0 months, *P* = 0.350) or stage IV (10.6 vs. 10.2 months, *P* = 0.828) patients (Fig. 1b, c).

Multivariable analysis using a Cox proportional hazards model showed that a PS ≥2 (HR = 2.75, 95% CI 1.71–4.38, *P* < 0.001) indicated a worse prognosis. An *EGFR* mutation (HR = 0.58, 95% CI 0.40–0.84, *P* = 0.004) and receiving systemic treatment (HR = 0.36, 95% CI 0.25–0.53, *P* < 0.001) were independent prognostic factors (Table 2). Neither the number (multiple vs. single, *P* = 0.225) nor site (bilateral vs. unilateral, *P* = 0.414) of SCLNs was associated with an unfavorable OS. Similarly, neither lymph node size (*P* = 0.906) nor fixed SCLN (*P* = 0.847) was associated with an unfavorable OS.

**Table 2:** Multivariable analysis for independent factors on overall survival

Variable	HR (95% CI)	*P* value
ECOG PS (≥2 vs. <2)	2.75 (1.71–4.38)	*P* < 0.001
EGFR (mutation vs. wild type)	0.58 (0.40–0.84)	*P* = 0.004
Systemic treatment (yes vs. no)	0.36 (0.25–0.53)	*P* = 0.001

Among evaluable patients, 11.8% (10/85) were observed to have local recurrence after biopsy in the excisional biopsy group, and 16.7% (3/18) of patients suffered local progression in the incisional biopsy group, although the difference was not statistically significant; *P* = 0.695.

Postoperative and intraoperative complications occurred in 26 patients (12.9%). There was no difference in the excisional (20/163, 12.3%) and incisional biopsy groups (6/39, 15.4%); *P* = 0.602. The most common complication was postoperative pain, which accounted for 61.5% (16/26). Most complications occurred on the first postoperative day. The incidence of intraoperative bleeding, which was the most dangerous complication, tended to favor the incisional (5.1%) rather than the excisional biopsy group (0.6%); however, the difference was not statistically significant (*P* = 0.096). Complications such as exudation and swelling favored the excisional biopsy group; however, these differences did not reach statistical significance (Table 3). One patient who received an excisional biopsy suffered a cerebral infarction on the first postoperative day, and his condition worsened; he lived only 2.9 months. Another patient in the excisional biopsy group suffered a wound infection and dehiscence; however, the wound healed after drainage and antibiotic treatment. No other serious adverse events accrued, and none died because of the supraclavicular lymphadenectomy.

**Table 3:** Complications in excisional biopsy group and incisional biopsy group

Complications	Excisional biopsy	Incisional biopsy	*P* value
	(*n* = 163)	(*n* = 39)	
Pain	12 (7.4%)	4 (10.3%)	0.518
Bleeding	1 (0.6%)	2 (5.1%)	0.096
Swelling	5 (3.1%)	0 (0.0%)	0.585
Exudation	5 (3.1%)	0 (0.0%)	0.585
Other^a^	3 (1.8%)	0 (0.0%)	1.000
Total	20 (12.3%)	6 (15.4%)	0.602

^a^Cerebral infarction (*n* = 1); fever (*n* = 1); wound infection (*n* = 1)

## Discussion

To our knowledge, this was the first study to explore the prognosis of SCLN-biopsied lung cancer patients. Patient outcomes suggested that SCLN incisional biopsies were safe and feasible and had no influence on the prognosis of advanced NSCLC patients.

Many factors might affect the prognosis of lung cancer. One of the most important factors is clinical stage [1]. This means that the survival of lung cancer mainly depends on the M and N stages for stages IV and IIIB patients, respectively. Collaud et al*.* found that the prognosis of lung cancer patients with microscopic residual tumor after resection was poorer than curative resection in stages I and II disease, but the difference was not observed in stage III or IV disease [11]. All patients in this study had stage IV or IIIB disease, with stage IV patients accounting for the majority in this study, which may be the main reason why SCLN incisional biopsies were not related to an unfavorable prognosis. Nevertheless, our study indicated that OS did not show any difference between the two groups for stage IIIB patients. Because of the small sample size of stage IIIB patients, further studies are necessary to confirm this result.

Patients who received incisional biopsy in our study tended to have SCLNs that were of a larger or fixed size and were bulky. Bulky or fixed multi-station N2 was defined as IIIA4 disease for which surgery was not indicated, and which was related to a poor prognosis [12]. Previous studies revealed that multiple station N1 or N2 lymph node metastasis was a poor prognostic factor compared with single lymph node metastasis in non-advanced lung cancer [13, 14]. There were no data for N3 lung cancer previously, although Johnson and colleagues found that lymph node number may affect prognosis in stage IIIB/IIIC colon cancer [15]. We found that both the number and size of SCLNs were not associated with an unfavorable OS in advanced NSCLC, nor was the SCLN site or fixed lymph nodes.

We also found that the incidence of complications was not significantly different between the two groups. The intraoperative bleeding in the incisional biopsy group (5.1%) seemed more common than that in the excisional biopsy group (0.6%), though statistical significance was not reached. This may be because there were more patients with bulky lymph nodes in the incisional biopsy group, which may have increased the surgical difficulty.

The choice of biopsy techniques in melanoma has been a controversial issue for decades. Much of the evidence has tended to show that incisional biopsies do not influence the prognosis of melanoma [2–4]. Despite this, an excisional biopsy was still recommended as the priority biopsy method in melanoma [16]. One important reason was that incisional biopsies increased the risk of underdiagnosis in melanoma and other tumors [17–19]. All patients enrolled in our study were confirmed pathologically as SCLN metastatic; therefore, we did not calculate the rate of underdiagnosis. However, it should be noted that we could obtain sufficient tissue from a resection biopsy rather than a needle biopsy for pathologic diagnosis and biomarker detection.

Another problem was that some investigators were concerned that incisional biopsies might increase the risk of cancer cell seeding and dissemination [20, 21]. Pisters et al. found that a positive surgical margin was an independent adverse prognostic factor for local recurrence and survival in soft tissue sarcomas [22]. Park et al. found that the major failure pattern for incomplete resection of NSCLC was distant metastasis, despite the postoperative radiotherapy and chemotherapy [23].Furthermore, investigators found that the CT-guide required for a biopsy in lung cancer could increase the risk of pleural seeding, though the incidence was low [24]. Our study showed that local progression (16.7%) in the incisional biopsy group was slightly higher than local recurrence (11.8%) in the excisional biopsy group, but the difference was not statistically significant. Nevertheless, further studies are warranted to explore whether SCLN incisional biopsies could lead to metastasis or dissemination in advanced lung cancer.

The main limitation of this study was its retrospective nature, so it was inevitable that some incomplete and/or censored data existed. In obtaining such results, it is not our purpose to advocate the use of incisional biopsies; we only provide evidence to show that this approach does not result in a worse prognosis. In clinical practice, supraclavicular lymph nodes with metastatic carcinoma should be removed as much as possible, but for some cases, wide resection range would increase the risk of complications such as bleeding, lymphatic fistula. Furthermore, since complete resection is mainly dependent on a surgeon’s experience, our results would benefit for the decision-making. Here, we suggest that incisional biopsies would be performed only if complete excision were infeasible or accompanied with higher risk of complications

## Conclusions

In conclusion, our results revealed for the first time that SCLN incisional biopsies do not negatively influence the prognosis of NSCLC patients. It is safe and feasible to perform an SCLN incisional biopsy as a last resort in lung cancer.

## Abbreviations

EGFR: Epidermal growth factor receptor
GLCI: Guangdong lung cancer institute
NSCLC: Non-small cell lung cancer
SCLN: Supraclavicular lymph node
